# Involvement of Oxidative Stress in the Development of Subcellular Defects and Heart Disease

**DOI:** 10.3390/biomedicines10020393

**Published:** 2022-02-07

**Authors:** Naranjan S. Dhalla, Vijayan Elimban, Monika Bartekova, Adriana Adameova

**Affiliations:** 1 St. Boniface Hospital Albrechtsen Research Centre, Institute of Cardiovascular Sciences, Department of Physiology and Pathophysiology, Max Rady College of Medicine, University of Manitoba, Winnipeg, MB R2H 2A6, Canada; VElimban@sbrc.ca; 2 Centre of Experimental Medicine, Institute for Heart Research, Slovak Academy of Sciences, Dubravska cesta 9, 84104 Bratislava, Slovakia; monika.bartekova@savba.sk (M.B.); adriana.duris.adameova@uniba.sk (A.A.); 3 Department of Pharmacology and Toxicology, Faculty of Pharmacy, Comenius University Bratislava, Odbojarov 10, 83232 Bratislava, Slovakia

**Keywords:** oxyradicals and oxidants, sarcolemmal membrane, sarcoplasmic reticulum, mitochondrial Ca^2+^-overload, myofibrillar Ca^2+^-sensitivity, subcellular Ca^2+^-handling

## Abstract

It is now well known that oxidative stress promotes lipid peroxidation, protein oxidation, activation of proteases, fragmentation of DNA and alteration in gene expression for producing myocardial cell damage, whereas its actions for the induction of fibrosis, necrosis and apoptosis are considered to result in the loss of cardiomyocytes in different types of heart disease. The present article is focused on the discussion concerning the generation and implications of oxidative stress from various sources such as defective mitochondrial electron transport and enzymatic reactions mainly due to the activation of NADPH oxidase, nitric oxide synthase and monoamine oxidase in diseased myocardium. Oxidative stress has been reported to promote excessive entry of Ca^2+^ due to increased permeability of the sarcolemmal membrane as well as depressions of Na^+^-K^+^ ATPase and Na^+^-Ca^2+^ exchange systems, which are considered to increase the intracellular of Ca^2+^. In addition, marked changes in the ryanodine receptors and Ca^2+^-pump ATPase have been shown to cause Ca^2+^-release and depress Ca^2+^ accumulation in the sarcoplasmic reticulum as a consequence of oxidative stress. Such alterations in sarcolemma and sarcoplasmic reticulum are considered to cause Ca^2+^-handling abnormalities, which are associated with mitochondrial Ca^2+^-overload and loss of myofibrillar Ca^2+^-sensitivity due to oxidative stress. Information regarding the direct effects of different oxyradicals and oxidants on subcellular organelles has also been outlined to show the mechanisms by which oxidative stress may induce Ca^2+^-handling abnormalities. These observations support the view that oxidative stress plays an important role in the genesis of subcellular defects and cardiac dysfunction in heart disease.

## 1. Introduction

Several clinical and experimental investigations have shown that oxidative stress plays a critical role in the pathogenesis of heart disease [1,2,3,4,5,6,7,8,9,10,11,12,13]. It is also becoming evident that the development of oxidative stress is invariably associated with the occurrence of cardiac dysfunction in different types of cardiovascular diseases such as atherosclerosis, myocardial infarction, hypertension, diabetes and various types of cardiomyopathies [14,15,16,17,18,19,20,21,22,23,24]. These studies have revealed that oxidative stress may induce cardiac remodeling, fibrosis, apoptosis, necrosis, metabolic defects and Ca^2+^-handling abnormalities in cardiomyocytes as well as endothelial dysfunction. Furthermore, various antioxidants have been observed to exert beneficial effects in improving cardiac function and attenuating heart disease [25,26,27,28,29,30,31]. The present article is focused on updating the existing information regarding the generation and implications of oxidative stress during the development of cardiovascular disorders. It is planned to emphasize the involvement of oxidative stress as a mechanism for inducing cardiac dysfunction as well as abnormalities in subcellular organelles and Ca^2+^-handling in cardiomyocytes in chronic myocardial infarction. Although oxidative stress is known to modify different proteins in subcellular organelles by affecting cardiac gene expression and various signal transduction mechanisms as well as by activating different proteases, no effort is made to deal with these issues in this article. On the other hand, some evidence is presented in this review to show that oxyradicals and some oxidants exert direct effects on subcellular organelles, and thus may explain the role of oxidative stress in the development of subcellular abnormalities, Ca^2+^-handling defects and cardiac dysfunction in heart disease.

## 2. Generation of Oxidative Stress in Heart Disease

It is now well known that the occurrence of oxidative stress is mainly a consequence of excessive formation of reactive oxygen species (ROS) including superoxide radicals and hydroxyl radicals as well as oxidants such as hydrogen peroxide (H_2_O_2_) and hypochlorous acid (HOCl) and/or reduction in the activities of endogenous antioxidants such as superoxide dismutase, glutathione peroxidase and catalase [1,32,33,34,35]. The transcription factors such as KLF9 (Kruppel-like factor 9) and Nrf2 (nuclear factor erythroid-2 related factor 2), which control the development of oxidative stress and the expression of antioxidant genes, have been reported to be activated in heart disease, respectively [36,37,38]. It should be pointed out that excessively produced nitric oxide (NO) due to the activation of NO synthase is known to combine with superoxide radicals to form peroxynitrite and produce nitrosative stress [39,40,41]. Furthermore, inflammatory cytokines such as tumor necrosis factor-alpha (TNF-α) have been shown to promote the development of both oxidative stress and nitrosative stress due to the activation of myeloperoxidase (for the generation of superoxide radicals) and NO synthase [42,43,44,45,46]. It is also noteworthy that the production of nitrosative stress is considered to be a major cause for the endothelial dysfunction in heart disease [47,48,49,50]. In fact, both oxidative stress and nitrosative stress have been demonstrated to activate the nuclear enzyme poly (ADP-ribose) polymerase to cause the fragmentation of DNA strands as well as initiate lipid peroxidation, protein oxidation and endothelial dysfunction in diseased myocardium.

Although ROS are considered to be mainly generated as a by-product of defects in mitochondrial metabolism and uncoupling of electron transport in heart disease, several enzymes such as xanthine oxidase, NADPH oxidase, nitric oxidase synthase, myeloperoxidase and monoamine oxidase are also involved in this process [51,52,53,54,55]. ROS production due to impaired electron transport in mitochondria is balanced by mitochondrial antioxidant enzymes such as superoxide dismutase and glutathione peroxidase but is augmented by different chronic pathological conditions [56,57,58,59]. Mitochondrial uncoupling proteins, which promote the leakage of protons across the inner mitochondrial membrane, are considered to be the promoters of mitochondrial ROS generation [58]. Furthermore, mitochondria were shown to integrate ROS signals from other cellular sources and promote the development of oxidative stress through a process termed as “ROS-induced ROS release” involving mitochondrial ion channels [59]. Increased myocardial fatty acid uptake has been shown to promote palmitoyl carnitine oxidation, increase formation of ROS and induce mitochondrial structural remodeling [60]. In addition, exposure of mitochondria for a prolonged period to palmitate was observed to enhance ROS generation associated with mitochondrial fission [60]. Angiotensin II has also been reported to promote ROS generation and produce mitochondrial DNA deletion as well as autophagy in cardiomyocytes [61]. In fact, chronic increase in ROS in mitochondria has been demonstrated to produce mitochondrial oxidative stress, which is associated with mitochondrial DNA damage [62,63]. Overexpression of mitochondrial transcription factor A (TFAM) and genes for mitochondrial antioxidant, peroxiredoxin-3 (PRX-3), were observed to attenuate ROS-induced mitochondrial oxidative stress, DNA deletion and decrease in oxidative phosphorylation activities [64,65].

The family of NADPH oxidase (NOX) are transmembrane proteins, which are involved in the generation of ROS by transferring electrons from NADPH to molecular oxygen, and serve as signaling molecules for inducing cardiac hypertrophy, apoptosis, fibrosis and heart failure [66,67,68]. ROS are released upon the activation of phagocytic NOS by acute myocardial infarction or reperfusion injury whereas abnormal stimulation of nonphagocytic NOS by angiotensin II, catecholamines and TNF-α has been implicated in cardiac hypertrophy collagen deposition, metalloprotease activation, fibrosis and heart failure [69]. The cascade of events triggered by increased activity of NOS includes lipid peroxidation and activation of mitogen-activated protein kinases (ERK 1/2, JNK and p38) for the occurrence of adverse cardiac remodeling [70]. It should be pointed out that NOX is present in different isoforms in multiple cell types; two isoforms (NOX2 and NOX4) are mainly expressed in cardiomyocytes [71,72,73,74]. The NOX2 isoform is localized in the sarcolemmal membrane and plays an important role in mediating angiotensin II-induced cardiac hypertrophy whereas the NOX4 isoform is localized in mitochondria, sarcoplasmic reticulum as well as nucleus and mediates adverse cardiac remodeling and heart failure due to pressure overload [72]. Inhibition of NOX2 was observed to prevent palmitate-induced abnormalities in mitochondrial respiration, ROS generation and Ca^2+^-overload [74]. Studies in mouse model of NOX4 knockout have revealed that NOX4 contributes to heart failure due to coronary ligation by increasing the inflammatory cytokine levels via enhancing the soluble epoxide hydrolase (a potent regulator of inflammation) [75], and in fact, NOX4 is a major source of mitochondrial ROS production in heart failure due to pressure-overload [76]. Apocynin, an inhibitor of NOX was found to attenuate oxidative stress, cardiomyocyte apoptosis and heart failure due to myocardial infarction in rabbits [77]. Furthermore, this agent ameliorated cardiac dysfunction, attenuated cardiac fibrosis and restored defects in Ca^2+^-handling activities of the sarcoplasmic reticulum in rabbits subjected to combined volume and pressure overload [78].

Another major source of ROS production is a monoamine oxidase (MAO), which participates in degradation of neurotransmitters such as norepinephrine, epinephrine and dopamine as well as serotonin [79,80]. This enzyme is localized in mitochondria and is involved in the production of H_2_O during the process of oxidative breakdown of catecholamines and serotonin. It should be noted that the levels of plasma catecholamines and serotonin are elevated in different types of heart disease and these may partly generate ROS during their degradation by MAO. In fact, MAO-A has been shown to play a key role in the development of acute and chronic heart diseases [81]. Exposure of cardiomyocytes to MAO-A has been reported to block autophagic flux with the accumulation of LC311, p62 and ubiquitylated proteins leading to mitochondrial fission and cellular necrosis [82]. Furthermore, the activation of MAO-A was found to result in the accumulation of lysosomal proteins (cathepsin D and Lamp 1), reduction in lysosomal acidification and blockade of the nuclear translocation of transcription factor-EB (TFEB), a regulator of autophagy and lysosome biogenesis [82]. It is also pointed out that MAO was found to be overactivated in ischemic heart disease [83], and its enhanced activity was shown to contribute to adverse cardiac remodeling and heart failure due to pressure overload [84]. Furthermore, the activation of MAO has been observed to depress mitochondrial function and result in heart failure as a consequence of oxidative stress in chronic diabetes [85].

## 3. Implications of Oxidative Stress in Heart Disease

**Figure 1:**
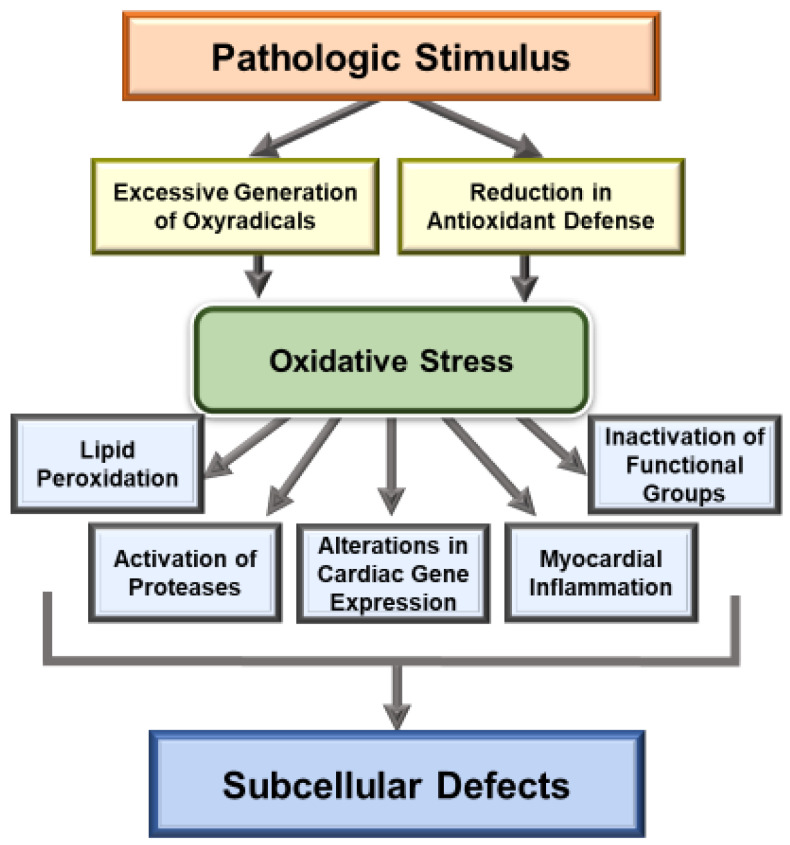
Mechanisms of oxidative stress induced defects in the function of subcellular organelles.

**Figure 2:**
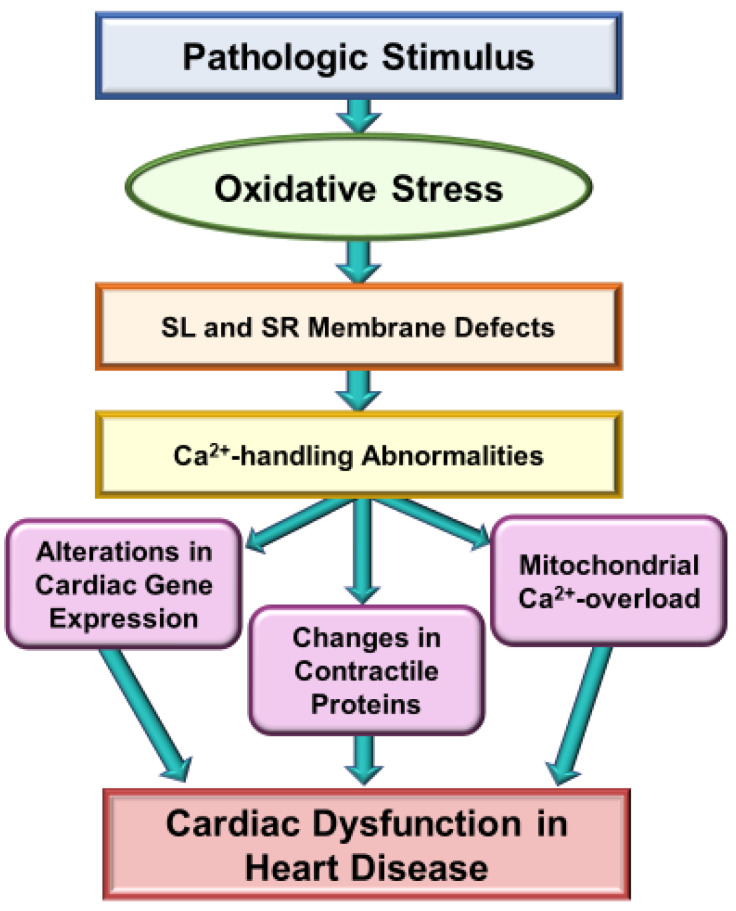
Role of Ca^2+^-handling abnormalities in oxidative stress induced cardiac dysfunction in heart disease. SL, sarcolemma; SR, sarcoplasmic reticulum.

Extensive research over the past several decades has revealed that oxidative stress may be a major mechanism for the genesis of cardiac dysfunction, subcellular defects and Ca^2+^-handling abnormalities in diverse cardiovascular disorders [1,86,87,88,89,90]. Such a role of both oxidative stress and nitrosative stress is mainly based on observations regarding the association of increased levels of various biomarkers of these stress factors and defects in subcellular organelles such as sarcolemma, sarcoplasmic reticulum, mitochondria and myofibrils [91,92,93,94]. Particularly, there occurs an increase in the level of inflammatory cytokines, depression in the activities of endogenous antioxidant enzymes and down-regulation of antioxidant defense mechanisms, including the Nrf2 pathway, which are known to promote the development of oxidative stress in diseased myocardium [1,35,38,42,43,44]. It is pointed out that the relationship of oxidative stress and inflammatory cytokines is of complex nature as both of these are known to promote the formation of each other in the diseased myocardium. Nonetheless, different antioxidant agents such as several vitamins, resveratrol and pterostilbene, as well as activation of the Nrf2-associated antioxidant pathway, have been shown to improve cardiac function, subcellular defects and Ca^2+^-handling abnormalities in heart disease [31,95,96,97,98,99]. Some of the major mechanisms for the induction of subcellular defects due to oxidative stress are depicted in Figure 1 and whereas those for the occurrence of Ca^2+^-handling abnormalities and cardiac dysfunction in heart disease are shown in Figure 2.

It is now well known that oxidative stress increases the intracellular Ca^2+^ in cardiomyocytes by promoting the entry of Ca^2+^ upon affecting the sarcolemmal membrane as well as by inducing changes in Ca^2+^-release and Ca^2+^-uptake activities in the sarcoplasmic reticulum under different pathological conditions [1,91]. Exposure of the heart to high levels of circulating angiotensin II or catecholamines for a prolonged period as well as in chronic myocardial infarction have been shown to induce abnormal Ca^2+^ -handling associated with mitochondrial Ca^2+^-overload, depression of mitochondrial function, generation of oxidative stress, activation of proteases, fragmentation of DNA and impaired cardiovascular function [1,91,95]. The loss of myofibrillar Ca^2+^-sensitivity due to prolonged oxidative stress and Ca^2+^-handling abnormalities is considered to explain cardiac dysfunction as a consequence of myofilament derangements and myofibrillar degeneration [91,99,100]. The oxidation of myofibrillar proteins such as actin, myosin and troponin has been shown to be accompanied by depressed ATPase activities as a consequence of oxidative stress [91,101]. Prolonged inhibition of xanthine oxidase was demonstrated to prevent myofibrillar oxidation and preserve cardiac function in a transgenic model of cardiomyopathy [102]. Failing hearts due to myocardial infarction exhibited a marked depression in myofibrillar Ca^2+^-stimulated ATPase activity due to the modification of myosin gene expression as a consequence of both oxidative stress and Ca^2+^-handling abnormalities in cardiomyocytes [91].

While excessive entry of extracellular Ca^2+^ through the sarcolemmal membrane has been shown to occur for the development of Ca^2+^-handling abnormalities in heart failure due to myocardial infarction, the exact contribution of Ca^2+^-influx and Ca^2+^-efflux mechanisms has not been fully established [91]. In this regard, the density of voltage dependent Ca^2+^-channels, which are associated with beat-to-heat Ca^2+^-influx through sarcolemma, was decreased but the activity of sarcolemmal Ca^2+^-pump, which is involved in Ca^2+^-efflux, was unaltered in the failing heart [1,91]. On the other hand, marked depression in sarcolemmal Na^+^-K^+^ ATPase in the infarcted heart has indicated that the Ca^2+^ entry in cardiomyocytes may increase indirectly through the Na^+^-Ca^2+^ exchange mechanism. In fact, the sarcolemmal Na^+^-Ca^2+^ exchange activity was also decreased, which may impair Ca^2+^-efflux (via the reverse mode) and contribute in the development of Ca^2+^-handling abnormalities in failing cardiomyocyte [103]. Furthermore, depression of the sarcolemmal Na^+^-K^+^ ATPase activity by cardiac glycosides has been shown to cause abnormal Ca^2+^-cycling and impair mitochondrial energetics in guinea pig cardiomyocytes [104]. Although oxidative stress can be seen to induce Ca^2+^-entry through the sarcolemmal membrane by increasing its permeability due to lipid peroxidation, changes in the sarcolemmal phospholipid composition by other mechanisms cannot be overlooked. Particularly, different oxyradical generating systems have been shown to decrease phospholipid N-methylation, which is known to determine the membrane fluidity [105]. Furthermore, oxidative stress has been demonstrated to modify the sarcolemmal activities of both phospholipases C and D, which may directly or indirectly participate in the occurrence of Ca^2+^-handling abnormalities in cardiomyocytes [91,106].

It needs to be emphasized that mitochondria is not only a major source for the production of cellular ATP but is also a major contributor for the production of oxyradicals in cardiomyocytes, and these processes are regulated by the intracellular concentration of Ca^2+^ [107,108]. Ca^2+^-handling abnormalities associated with oxidative stress are known to induce mitochondrial Ca^2+^-overload and impair mitochondrial function for the production of energy [109,110,111]. On the other hand, an increase in the mitochondrial ATPase inhibitory factor-1 due to oxidative stress has been shown to disrupt mitochondrial Ca^2+^-handling whereas a loss of mitochondrial Ca^2+^ uniporter has been reported to trigger arrhythmias possibly by affecting the Ca^2+^-handling function of the sarcoplasmic reticulum [112,113]. It is pointed out that the sarcoplasmic reticulum, by virtue of its ability to release and accumulate Ca^2+^ on a beat-to-heat basis, is known to play a major role in Ca^2+^-handling in cardiomyocytes, and has been indicated to serve as a critical target for oxidative stress. Both oxidative stress and nitrosative stress have been demonstrated to promote Ca^2+^-leak by modifying ryanodine receptors in the sarcoplasmic reticulum in various types of failing hearts [114,115,116]. Alterations in the sarcoplasmic reticulum Ca^2+^-pump ATPase and ryanodine receptors in heart failure are considered to occur due to oxidative stress as these were corrected by an antioxidant, edaravone, as well as by carvedilol (due to its antioxidant properties) [117,118].

## 4. Evidence for the Direct Action of Oxidative Stress on Subcellular Organelles

Although the development of oxidative stress has been shown to be associated with subcellular defects for the occurrence of Ca^2+^-handling abnormalities and subsequent cardiac dysfunction, it is not clear whether these effects of oxidative stress on subcellular organelles are of any direct or indirect nature. In order to gain some information in this regard, sarcolemmal membranes, sarcoplasmic reticulum, mitochondria and myofibrils were isolated from control hearts; these subcellular organelles were incubated with different oxyradical generating systems and oxidants for 30 min and their activities were determined. The results in Table 1 indicate that the incubation of sarcolemma with superoxide radical generating mixture, H_2_O_2_ and hydroxyl radical generating mixture depressed Na^+^-K^+^ ATPase and Na^+^-Ca^2+^ exchange activities. These changes were associated with increased malondialdehyde (MDA) content and reduced sulfhydryl groups (SH-groups). The effects of superoxide radicals were attenuated by superoxide dismutase (SOD), the effects of H_2_O_2_ were attenuated by catalase and those of hydroxyl radicals were reduced by mannitol [119,120]. The sarcolemmal ATP-dependent Ca^2+^ uptake activity, Ca^2+^-stimulated ATPase activity and Mg^2+^-ATPase activity were also depressed by superoxide radicals, H_2_O_2_ and hydroxyl radicals and these effects were attenuated by their scavengers, SOD, catalase and mannitol, respectively (Table 2) [120]. Furthermore, the density, unlike the affinity of Ca^2+^-binding and both low and high affinities of ATP-binding, were depressed whereas the activity of Ca^2+^-ecto ATPase (which serves as a Ca^2+^-gating mechanism) was increased by superoxide radicals, H_2_O_2_ and hydroxyl radicals (Table 3) [121,122]. Superoxide radicals and H_2_O_2_ were also observed to depress the sarcoplasmic reticulum Ca^2+^-release, Ca^2+^-uptake and Ca^2+^-pump ATPase activities whereas the myofibrillar Ca^2+^-stimulated ATPase activity and SH-group content were reduced and Mg^2+^-ATPase activity was increased (Table 4) [123,124,125]. Furthermore, both superoxide radicals and H_2_O_2_ depressed mitochondrial state 3 respiration, RCI value and ADP-to-O ratio, indicating impaired mitochondrial function (Table 4) [126]. The effects of incubating sarcolemma and myofibrils with a potent oxidant, HOCl, are shown in Table 5 [125,127]. The depressions in sarcolemmal Na^+^-K^+^ ATPase as well as SH-group content and increase in MDA content by HOCl were attenuated by the presence of its scavenger, methionine [127]. Furthermore, HOCl was observed to increase myofibrillar Mg^2+^-ATPase and decrease Ca^2+^-stimulated ATPase activities, these effects of HOCl were attenuated by methionine [125]. It is pointed out that most of the changes in subcellular activities induced by different oxyradical generating systems and oxidants under in vitro conditions are similar to those seen in failing hearts due to chronic myocardial infarction. Furthermore, the present in vitro observations indicate that the activities of different subcellular organelles are affected directly by oxyradicals and oxidants, thus, support the view that oxidative stress can induce subcellular organelles and Ca^2+^-handling abnormalities in cardiomyocytes for the occurrence of cardiac dysfunction in heart disease.

## 5. Concluding Remarks

An in-depth analysis of the literature regarding the pathophysiology and pharmacotherapy of different types of cardiovascular diseases has revealed that oxidative stress is one of the most critical factors, which is involved in the pathogenesis of cardiac dysfunction. It is also evident that the occurrence of oxidative stress represents a disbalance between the excessive formation of oxyradicals and the activities of antioxidant defense mechanisms. Furthermore, it has been demonstrated that the generation of oxyradicals in diseased myocardium is a consequence of defects in the mitochondrial electron transport system as well as the by-product of reactions involving different enzymes including NADPH oxidase, xanthine oxidase, nitric oxide synthase and monoamine oxidase. Although the contribution of each source to produce oxidative stress is not clear, it seems that the involvement of each source for the generation of oxyradicals may differ from one disease to the other and may be specific for the stage and type of heart disease. In addition, oxidative stress has been shown to produce a wide variety of changes in various metabolic pathways and signal transduction systems for the induction of fibrosis, necrosis and apoptosis as well as the depression of cardiac gene expression and activation of different proteolytic enzymes in heart disease. However, alterations in subcellular organelles such as sarcolemma, sarcoplasmic reticulum, mitochondria and myofibrils may be more related to the development of Ca^2+^-handling abnormalities and cardiac dysfunction due to oxidative stress in heart disease. Particularly, there occurs an excessive entry of Ca^2+^ due to increased membrane permeability (as a consequence of changes in lipid composition and activation of the Ca^2+^-gating mechanism) as well as depression in the sarcolemmal Na^+^-K^+^ ATPase and Na^+^-Ca^2+^ exchange systems due to oxidative stress. Furthermore, oxidative stress promotes the release of Ca^2+^ (due to defect in ryanodine receptors) and depresses Ca^2+^-uptake (due to changes in Ca^2+^-pump ATPase) in the sarcoplasmic reticulum. Such Ca^2+^-handling alterations in both sarcolemma and sarcoplasmic reticulum can be seen to induce mitochondrial Ca^2+^-overload and depress the process of energy production. Prolonged Ca^2+^-handling abnormalities in diseased myocardium will also cause derangements of myofilaments and loss of myofibrillar Ca^2+^-sensitivity. Taken together, all these defects in subcellular organelles can be seen to result in cardiac dysfunction due to the development of oxidative stress in heart disease. It should be emphasized that it is not our intention to exclude the participation of several other pathogenic factors in the pathogenesis of cardiac dysfunction but the information in this article provides evidence in support of the concept that oxidative stress may induce cardiac dysfunction due to subcellular defects and Ca^2+^-handling abnormalities in heart disease.

## Figures and Tables

**Table 1:** Modification of Na^+^-K^+^ ATPase and Na^+^-Ca^2+^ exchange activities as well as malondialdehyde (MDA) and sulfhydryl (SH-group) content of cardiac sarcolemma upon incubation for 30 min with or without different oxyradical generating systems.

Parameters	Control	X + XO	0.5 mM H_2_O_2_	0.1 mM H_2_O_2_ + 0.05 mM Fe^2+^
Na^+^-K^+^ ATPase (µmol Pi/mg/h)	14.27 ± 1.07	6.81 ± 1.03 *	8.64 ± 1.04 *	6.98 ± 0.13 *
Na^+^-Ca^2+^ exchange (nmol/mg/2 s)	4.59 ± 0.08	1.97 ± 0.13 *	2.97 ± 0.11 *	3.02 ± 0.14 *
MDA content (nmol/mg protein)	61.67 ± 3.67	81.85 ± 2.54 *	78.77 ± 3.23 *	88.26 ± 3.07 *
SH-group content (nmol/mg protein)	72.61 ± 2.37	37.59 ± 4.41 *	42.36 ± 2.75 *	40.86 ± 3.54 *

The data are taken from our papers (Kaneko et al. [119], and Kaneko et al. [120]). X + XO, 2 mM xanthine plus 0.03 U xanthine oxidase. The mixture of X+XO was used to generate superoxide radicals and the mixture of low concentrations of H_2_O_2_ and Fe^2+^ was used to generate hydroxyl radicals. * *p* < 0.05 vs. respective control.

**Table 2:** Modification of ATP-dependent Ca^2+^ accumulation, Mg^2+^ ATPase and Ca^2+^-stimulated ATPase activities in cardiac sarcolemma upon incubation for 30 min with different oxyradical generating systems in the absence or presence of their scavengers.

Parameters	ATP-Dependent Ca^2+^ Accumulation (nmol Ca^2+^/mg/5 min)	Mg^2+^ ATPase (µmol/mg/h)	Ca^2+^-Stimulated ATPase (µmol/mg/h)
Control	27.0 ± 1.7	195 ± 3	13.6 ± 0.7
X + XO treated	9.5 ± 0.8 *	176 ± 2 *	2.6 ± 0.5 *
X + XO + 80 µg/mL SOD	21.8 ± 0.8 †	192 ± 2 †	10.1 ± 0.4 †
0.5 mM H_2_O_2_ treated	4.7 ± 1.3 *	165 ± 6 *	2.9 ± 0.4 *
0.5 mM H_2_O_2_ + 10 µg/mL catalase	20.6 ± 1.1 †	190 ± 5 †	8.4 ± 0.3 †
0.1 mM H_2_O_2_ + 0.2 mM Fe^2+^ treated	6.7 ± 0.5 *	169 ± 4 *	4.0 ± 0.3 *
0.1 mM H_2_O_2_ + 0.2 mM Fe^2+^ + 20 mM mannitol	17.2 ± 0.8 †	184 ± 2 †	8.0 ± 0.5 †

The data are taken from our paper (Kaneko et al. [120]). X + XO, 2 mM xanthine oxidase plus 0.03 U/mL xanthine oxidase. The mixture of X + XO was used to generate superoxide radicals whereas that with low concentration of H_2_O_2_ plus Fe^2+^ was used for generating hydroxyl radicals. SOD, superoxide dismutase. * *p* < 0.05 vs. respective control, † *p* < 0.05 vs. respective oxyradical treated.

**Table 3:** Modification of Ca^2+^-channels, ATP receptors, Ca^2+^-binding and Ca^2+^-ecto ATPase in cardiac sarcolemma upon incubation for 30 min with oxyradical generating systems.

Parameters	Control	X+XO	1 mM H_2_O_2_	0.1 mM H_2_O_2_ + 0.2 mM Fe^2+^
**A. Ca^2+^-channel binding**				
Kd (nM)	0.231 ± 0.011	0.252 ± 0.011	0.254 ± 0.018	0.267 ± 0.017
Bmax (fmol/mg)	199 ± 12	139 ± 7.0 *	142 ± 8.0 *	157 ± 9.0 *
**B. ATP-binding**				
Low affinity (1.25 mM Ca^2+^)	97.8 ± 4.3	147.2 ± 6.1 *	141.3 ± 5.4 *	41.4 ± 4.9 *
High affinity (50 µM Ca^2+^)	7.95 ± 0.32	12.08 ± 0.68 *	13.92 ± 0.66 *	4.08 ± 0.24 *
**C. Ca^2+^-ecto ATPase**				
(µmol Pi/mg/h)	44.3 ± 1.1	57.7 ± 1.4 *	57.0 ± 1.2 *	31. 4 ± 1.3 *

The data are taken from our papers (Kaneko et al. [121] and Kaneko et al. [122]). X + XO, 2 mM xanthine plus 0.03 U xanthine oxidase. The mixture of X + XO was used to generate superoxide radicals whereas the mixture of H_2_O_2_ plus Fe^2+^ mixture was used for the generation of hydroxyl radicals. * *p* < 0.05 vs. respective control.

**Table 4:** Modification of some biochemical activities of cardiac sarcoplasmic reticulum, myofibrils and mitochondria upon incubation for 30 min with some oxyradical generating systems.

Parameters	Control	X+XO	1 mM H_2_O_2_
**A. Sarcoplasmic reticulum:**			
Ca^2+^-release (nmol Ca^2+^/mg/15 s)	8.5 ± 1.4	4.2 ± 0.8 *	3.9 ± 0.7 *
Ca^2+^-uptake (nmol Ca^2+^/mg/min)	29.6 ± 2.4	15.9 ± 1.6 *	12.7 ± 1.5 *
Ca^2+^-pump ATPase (µmol Pi/mg/h)	14.7 ± 1.3	6.4 ± 0.8 *	5.7 ± 0.9 *
**B. Myofibrils:**			
Mg^2+^-ATPase (µmol/mg/h)	2.53 ± 0.13	4.97 ± 0.16 *	5.46 ± 0.18 *
Ca^2+^-stimulated ATPase (µmol/mg/h)	10.29 ± 0.17	6.48 ± 0.18 *	5.92 ± 0.38 *
Sulfhydryl group content (nmol/mg protein)	67.0 ± 1.3	54.2 ± 1.6 *	47.6 ± 2.06 *
**C. Mitochondria**			
State 3 respiration (O/mg/min)	293 ± 7.0	138 ± 7.0 *	106 ± 4.0 *
RCI (State 3 to state 4 ratio)	5.36 ± 0.13	2.66 ± 0.23 *	1.89 ± 0.07 *
ADP to O ratio (nmol ADP/ng atom O)	3.00 ± 0.15	2.55 ± 0.07 *	2.37 ± 0.03 *

The data are taken from our papers (Matsubara and Dhalla [123], Takeda et al. [124], Suzuki et al. [125] and Makazan et al. [126]). X + XO, 2 mM xanthine plus 0.03 U xanthine oxidase. This mixture was used to generate superoxide radicals. * *p* < 0.05 vs. respective control.

**Table 5:** Modification of ATPase activities in cardiac sarcolemma and myofibrils upon incubation with HOCl for 30 min in the presence or absence of L-methionine.

Parameters	Control	0.1 mM HOCl	HOCl Plus 10 mM L-methionine
**A. Sarcolemma:**			
Na^+^-K^+^ ATPase (µmol Pi/mg/h)	18.86 ± 2.03	2.16 ± 1.05 *	13.31 ± 2.44 †
MDA content (nmol/mg protein)	51.64 ± 3.97	67.33 ± 3.97 *	48.2 ± 3.59 †
Sulfhydryl group content (nmol/mg protein)	64.84 ± 6.36	28.67 ± 4.40 *	55.86 ± 5.72 †
**B. Myofibrill ATPase** **(µmol/mg/h)**			
Mg^2+^ ATPase	2.80 ± 0.12	9.51 ± 0.16 *	3.79 ± 0.16 †
Ca^2+^-stimulated ATPase	10.96 ± 0.15	5.73 ± 0.31 *	11.64 ± 0.12 †

The data are taken from our papers (Kato et al. [127] and Suzuki et al. [125]). MDA, malondialdehyde. * *p* < 0.05 vs. respective control; † *p* < 0.05 vs. respective HOCl.
